# Antioxidant Capacity of Cultured Mammalian Cells Estimated by ESR Method

**DOI:** 10.1100/tsw.2004.99

**Published:** 2004-06-29

**Authors:** Tamar Kartvelishvili, Marina Abuladze, Nino Asatiani, Joseph Akhvlediani, Lali Asanishvili, Hoi-Ying N. Holman, Nelly Sapojnikova

**Affiliations:** ^1^ Institute of Molecular Biology and Biophysics, Georgian Academy of Sciences, 14,Gotua Str., 0160 Tbilisi, Georgia; ^2^ Institute of Physics, Georgian Academy of Sciences, 6, Tamarashvili Str., 0177 Tbilisi, Georgia; ^3^ Center for Environmental Biotechnology, E.O. Lawrence Berkeley National Laboratory, 1, Cyclotron Road, Berkeley, CA, 94720, USA

**Keywords:** antioxidant systems, cell culture, ESR

## Abstract

In the present study, the antioxidant capacity against hydrogen peroxide (H_2_O_2_), one of the stress-inducing agents, was investigated in two distinct cell lines: L-41 (human epithelial-like cells) and HLF (human diploid lung fibroblasts), which differ in tissue origin, life span in culture, proliferate activity, and special enzyme system activity. The cell antioxidant capacity against H_2_O_2_ was estimated by the electron spin resonance (ESR) spin-trapping technique in the Fenton reaction system via Fe^+2^ ion action with H_2_O_2_ resulting in hydroxyl radical generation. The effects of catalase inhibitors, such as sodium azide and 3-amino-1,2,4-triazole, on the antioxidant capacity of cells were tested. Based on our observation, it can be concluded that the defensive capacity of cells against H_2_O_2_ depends on the ratio between catalase/GPx/SOD and H_2_O_2_, especially at high-stress situations, and the intracellular balance of these enzymes are more important than the influence of the single component.

## INTRODUCTION

Oxygen utilization is a general consequence of aerobic life. Oxygen forms different intermediates of its reduction such as superoxide anion ($O_2^-$), hydrogen peroxide ($H_2O_2$), singlet oxygen ($O_2^1$), ·OH radicals (mean reactive oxygen species [ROS]), which are products of normal cellular metabolism, and a variety of exogenous stress-inducing agents as well.

The mitochondrion is the organelle in eukaryotic cells responsible for aerobic respiration and it is the most common source of ROS. In normal cells, 1–2% of electrons carried by the mitochondrial electron transport chain leak from this pathway and pass directly to oxygen-generating superoxide anion, which can be a source of other ROS[1].

ROS are involved in cell growth, differentiation, and death. Low concentrations of ROS may be beneficial for living organisms. On the other hand, since ROS can react with DNA, proteins, carbohydrates, and lipids in a destructive manner and cause oxidative stress, the higher concentrations of ROS may play a role in aging and in a number of human diseases[2,3,4,5].

Radical chain termination can be caused by the interaction of radicals with different antioxidants (glutathione [GSH], ascorbic acid, uric acid, $\alpha$-tocopherol, etc.) and special enzyme systems as well. The cell possesses extensive ubiquitous and most effective pathways against ROS. Superoxide can be readily converted by mitochondrial (Mn-SOD) and cytosolic (Cu,Zn-SOD) superoxide dismutases into $H_2O_2$, one of the stress-inducing agents potentially capable of forming an oxidative intermediate in cells; can be decomposed primarily by catalase ($H_2O_2 \rightarrow H_2O + O_2$) (in the peroxisomes) and by glutathione peroxidase (GPx) ($H_2O_2 \rightarrow H_2O$) (in the cytoplasm and mitochondria) via GSH dependent cycle, which is a mechanism in scavenging alkylhydroperoxides; and is complementary to catalase in scavenging $H_2O_2$[1,5,6].

Pathology-induced conditions, which increase the rate of $H_2O_2$ production or deplete components of antioxidant systems, e.g., GSH, will lead to accumulation of $H_2O_2$ in the cytosol or mitochondria. In biological systems, $H_2O_2$ could readily diffuse across cellular membranes and lead to depletion of ATP, GSH, and NADPH. By scission of the peroxide bond through the Fenton reaction[1,7], hydrogen peroxide could generate hydroxyl radical, an extremely potent oxidant, which is able to cause damage of most biological macromolecules. When ROS overcome the defense systems of the cell and redox homeostasis is altered, the result is an oxidative stress.

The aim of the present study was the estimation of the constitutive antioxidant capacity against $H_2O_2$ investigated in two distinct cell lines: normal diploid cells (HLF: human diploid lung fibroblasts) and immortalized cell line L-41 (human epithelial-like cells).

## METHODS

### Cell Culture

The L-41 was derived from the J-96 cell line originally obtained from a patient with monocytic cell leukemia (Research Center of Medical Genetics, Russian Academy of Medical Science, Moscow)[8,9]. The L-41 cells were maintained as adherent cells in Eagle's culture medium supplemented with 10% donor calf serum, 2 m*M* L-glutamine, 100 units of penicillin/ml, and 100 µg of streptomycin/ml at 37ºC in a 5% $CO_2$ incubator. Cells were harvested with trypsin (0.25%)/EDTA solution. The cells seeded at the concentration $1 \times 10^5$/ml are confluent in 3 days.

The HLF cells were maintained as adherent cells in Dulbecco's modified Eagle's culture supplemented with 15% fetal bovine serum, 2 m*M* L-glutamine, 100 units of penicillin/ml, and 100 µg of streptomycin/ml at 37ºC in a 5% $CO_2$ incubator. Cells were harvested with trypsin (0.25%)/EDTA solution. Cells seeded at a concentration of $3 \times 10\ ^5$/ml are confluent in 7 days.

L-41 and HLF cell lines differ in tissue origin, life span in culture, and proliferate activity. The mitotic index (Mi,$^o/_{oo}$) of L-41 cells reaches about 35–40$^o/_{oo}$ at 48 hr of cultivation, while the mitotic index of HLF does not exceed 20$^o/_{oo}$ for the same time.

The antioxidant capacity of both cell lines was estimated when cells were in an exponential growth phase and achieved 80% of confluence.

### ESR Measurements

The electron spin resonance (ESR) spin-trapping technique was employed to detect short-lived ·OH radicals in model Fenton reaction. ·OH radicals were trapped by 5,5-dimethyl-1-pyrroline N-oxide

(DMPO, Sigma). ESR spectra were obtained by using ESR spectrometer RE 1306 (Russia) at 100 kHz modulator frequency, 1.2G modulation amplitude, 25 m*M* microwave power, microwave frequency 9.3 GHz. The samples were placed in the ESR cavity using cell-glass capillaries with an internal diameter of 1 mm. Spectra were recorded at ambient temperature. The ESR spectra consist of 1:2:2:1 quartet with splitting of $a_H = a_N = 14.8$ G, where $a_N$ and $a_H$ denote hyperfine splitting of the nitroxyl nitrogen and β-hydrogen, respectively, which are typical for this system[10,11]. The ratio of the second signal intensity of DMPO/·OH adduct to the intensity of the reference (DPPH-1,1-diphenyl-2-picryl-hydrazyl as a reference) was estimated and presented as arbitrary units (A.U.) in the Figures. The reaction mixture contained 100 m*M* DMPO, 1 m*M* $FeSO_4$, 100 m*M* $H_2O_2$, 50 m*M* sodium-potassium buffer pH 7.4 in a final volume 62.5 µl.

## Glutathione Antioxidant System

Glutathione concentration was determined by using GSH-400 colorimetric assay (Oxis, USA). Glutathione reductase (GR) activity was measured by using the BIOXYTECH GR-340™ Assay (Oxis, USA). Glutathione peroxidase (GPx) activity was determined by using BIOXYTECH GPx-340™ colorimetric assay for cellular glutathione peroxidase (Oxis, USA).

## Cellular Superoxide Dismutase (SOD)

The technique of SOD assay involves photoreduction of nitro blue tetrazolium (NBT) for the determination of activity of SOD following native polyacrylamide gel (PAAG) electrophoresis. The protein corresponding to SOD can be then visualized as an achromatic zone through the inhibition of NBT (Sigma) reduction via SOD[12]. Achromatic bands were visualized for 50 µg protein equivalent. Protein concentration in the both cell extracts was determined using BCA (bicinchoninic acid) protein assay reagent (Pierce, USA). The fractionated proteins were separated by SDS-PAAG and native PAAG electrophoresis and stained with Coomassie blue. Coomassie-blue-stained gels show equal amounts of protein loaded.

The positions of the two isozymes of SOD, Cu,Zn-SOD and Mn-SOD, were identified by incubation of the cell lysate at 37ºC with 2% (w/v) sodium dodecyl sulfate (SDS) for selective inactivation of Mn-SOD[13]. After incubation, the upper achromatic zone was identified as Mn-SOD and the lower as Cu,Zn-SOD.

## Catalase Activity

Catalase activity in the cell crude extract was determined by measuring the rate of $H_2O_2$ (10 m*M*) decomposition in 50 m*M* potassium phosphate buffer, (pH 7.0), in the presence of the cell crude extract at 240 nm and 25°C, $\varepsilon_{H2O2} = 43.6\ M^{-1}\ cm^{-1}$[14].

## Preparation of the Cell Crude Extract

The catalase and SOD activities were investigated in cell crude extracts. Cells ($\sim 10^7$) were harvested by centrifugation (3,000 rpm, 5 min, 4ºC), rinsed twice in 50 m*M* phosphate buffer, pH 7.8. The rinsed cells were resuspended in a definite volume of above-mentioned buffer 1:4 (w/v), sonicated five times for 10-sec bursts (44 kHz), centrifuged (14,000 rpm, 20 min, 4ºC), and the soluble extract was used as a sample. The cell crude extracts were standardized per microgram of total protein. Protein concentrations in the cell extract were determined using BCA protein assay reagent (Pierce, USA).

## RESULTS AND DISCUSSION

### Estimation of Defense System Activities

The defense system activities were estimated for two generally used culture models: (1) normal diploid, nontumorigenic cells with limited life span susceptible to senescence (HLF cells); (2) immortalized cell line with stable phenotype in cell culture, high proliferate capacity with extended life span through mechanisms increasing genomic instability (L-41 cells).

In contrast with the normal HLF cells, L-41 have significantly higher levels of catalase and GSH cycle (Table 1), which includes GSH-cofactor of the GPx. GPx is the enzyme that oxidizes GSH to GSSG and glutathione reductase (GR) is the enzyme that regenerates GSH from GSSG. Hence, the selected cell lines have distinct defensive systems against $H_2O_2$.

**TABLE 1**
**Catalase and GSH Cycle Enzyme Activities of the Cells**

| Cell Type | Catalase (U/ml) | GSH (μM) | GR (mU/ml) | GPx (mU/ml) |
|---|---|---|---|---|
| L-41 | 217 ± 23 | 863 ± 89 | 100 ± 15 | 82 ± 12 |
| HLF | 84 ± 12 | 100 ± 14 | 41 ± 8 | 54 ± 8 |

Each value is the mean ± S.D. from three separate sets of experiments. Significant difference in the enzyme activities and glutathione quantity ($p < 0.05$, Student's *t*-test) was obtained between the studied cell lines.

It should be noted that in contrast to L-41 cells, HLF cells are characterized by higher constitutive activities of Mn- and Cu,Zn-SOD (Fig. 1).

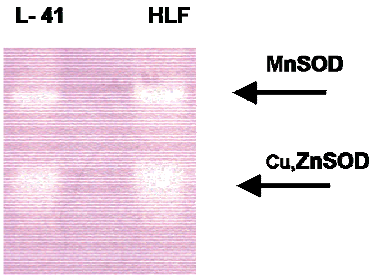

**FIGURE 1.** The photochemical NBT method of SOD detection in 12% native gel. 50 μg of the total protein has been applied to the gel.

### Estimation of the Cell Antioxidant Capacity by ESR

The intracellular antioxidant capacity of cells was estimated by the ESR spin-trapping technique in an artificial Fenton reaction system, a common source of •OH radicals, via $Fe^{+2}$ ion action with $H_2O_2$. Hydroxyl radicals were trapped by DMPO.

The ESR spectra consist of 1:2:2:1 quartet with splitting of $a_H = a_N = 14.8$ G, where $a_N$ and $a_H$ denote hyperfine splitting of the nitroxyl nitrogen and β–hydrogen, respectively, which are typical for this system[10,11].

The antioxidant capacity of cells was registered for the definite periods of time after the addition of the cell crude extracts to the model Fenton reaction. The time-dependent generation of •OH radicals in the model Fenton reaction trapped by DMPO is presented in Fig. 2.

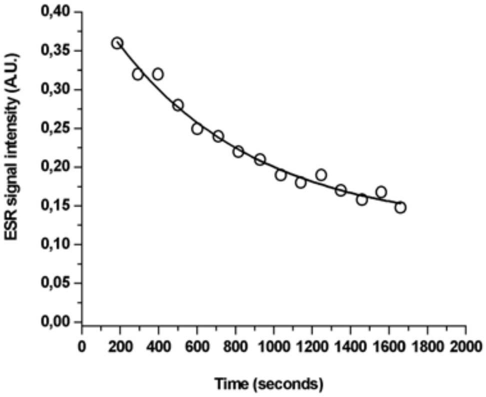

**FIGURE 2.** The time course of the ESR signal intensity in model Fenton reaction. The model Fenton reaction mixture contained 100 m*M* DMPO, 1 m*M* $FeSO_4$, 100 m*M* $H_2O_2$, 50 m*M* sodium/potassium buffer pH 7.4 in a final volume 62.5 µl.

Both cell crude extracts provoked the inhibition of DMPO/•OH spin-adduct signal, but to different extent. As it follows from Fig. 3, L-41 cells, which catalase, and GPx activities are higher than in HLF cells, suppress the model Fenton reaction more actively via decomposition of $H_2O_2$, taking into account as initial as well final steps of reaction (Fig. 3).

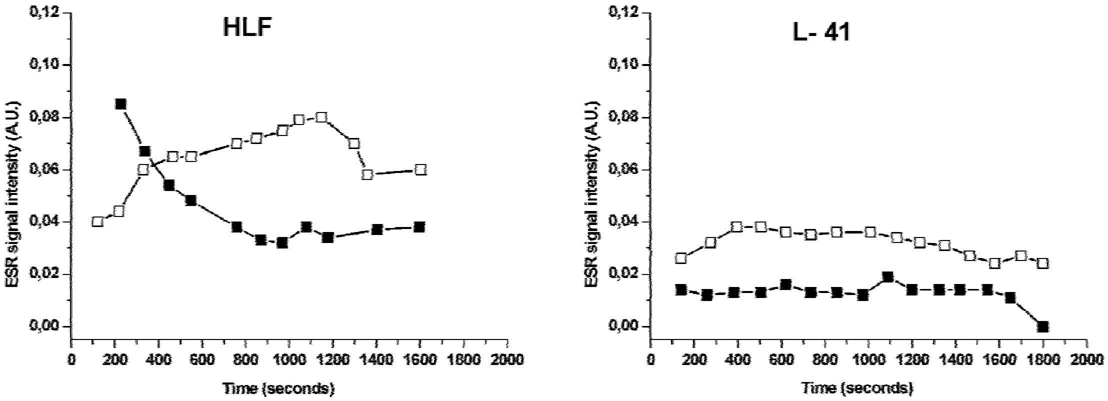

**FIGURE 3.** The time course of the ESR signal intensity in model Fenton reaction in the presence of L-41 and HLF cell crude extracts. ■ – 1 × 0.168 mg/ml of protein in the ESR sample; □ – 2 × 0.168 mg/ml of protein in the ESR sample. The model Fenton reaction mixture contained 100 m*M* DMPO, 1 m*M* $FeSO_4$, 100 m*M* $H_2O_2$, 50 m*M* sodium/potassium buffer pH 7.4 in a final volume 62.5 µl.

The twofold increase of protein concentration (2 × 0.168 mg/ml) in the studied ESR samples causes twofold increase of catalase and GPx quantities, i.e., activities, and as a result should increase antioxidant capacity. In spite of the expectation, the increase of protein quantity has excited the increase of the ·OH radical levels (diminution of the inhibitory capacity of the cell crude extracts against $H_2O_2$).

## Estimation of Inhibitors Action

It was reported that in the presence of $H_2O_2$, catalase was reduced by the addition of sodium azide ($NaN_3$) and 3-amino-1,2,4-triazole (3-AT)[15,16].

The effects of catalase inhibitors, such as $NaN_3$ and 3-AT, on the antioxidant capacity of cell crude extracts were investigated. These inhibitors differed in their effects on enzymes. $NaN_3$ inactivates catalase by directly attaching to the coordination position of the iron in the heme moiety and thus inhibits subsequent reaction with $H_2O_2$ to produce an inactive ferrous derivative[15,16]. In contrast, 3-AT was found to be a markedly less potent inhibitor binding to the protein part of the catalase. It was documented that the action of 3-AT is quite slow[15,16]. In our study, the action of 3-AT was relatively weak under the same conditions of the measurement. In both cells (HLF and L-41), $NaN_3$ and 3-AT added to the cell crude extracts excited the increase of ·OH radical levels via inhibition of the enzymes, at $1 \times 0.168$ mg/ml and $2 \times 0.168$ mg/ml total protein concentrations in ESR sample mixture (Fig. 4). The effect of $NaN_3$ on cell crude extracts was much higher than the effect of 3-AT, which can be caused by low inhibitory capacity of 3-AT[16].

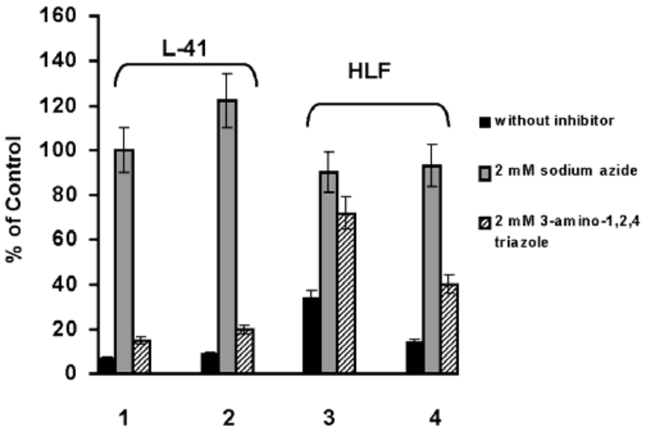

**FIGURE 4.** Effects of $NaN_3$ and 3-AT inhibitory action on the antioxidant capacity of cell crude extracts estimated by ESR method. ·OH-radical level in the model Fenton reaction is assigned as 100%. ESR measurements were conducted in 120 sec after preparation of the mixture; (1) and (3) are $1 \times 0.168$ mg/ml of protein in the ESR sample; (2) and (4) are $2 \times 0.168$ mg/ml of protein in the ESR sample. The data presented are mean values ± S.D. from three separate sets of experiments.

The action of $NaN_3$ on cell crude extracts was different at $1 \times 0.168$ mg/ml and $2 \times 0.168$ mg/ml total protein concentrations. In the latter case the level of ·OH radicals exceeded the model system level in L-41 cells. The effect of 3-AT was more active in HLF cells compared with L-41 cells (Fig. 4).

## Effect of the Cu,Zn-SOD Action

Generally superoxide dismutases (michondrial Mn-SOD and cytosolic Cu,Zn-SOD) catalize superoxide dismutation resulting in $H_2O_2$ generation[17]. It was documented that SOD can induce a toxic effect[18,19]. The reasons for the toxicity of Cu,Zn-SOD can be the increase of $H_2O_2$ levels[19], which is insufficiently decomposed by catalase and/or the increase of ·OH radical level in presence $Fe^{+2}$ and $Cu^{+1}$ ions via Fenton or Fenton-like reaction, respectively[20,21,22]. In contrast to mitochondrial Mn-SOD, which does not react with $H_2O_2$[23,24], cytosolic Cu,Zn-SOD, which reacts through the cyclic reduction and oxidation of the bound copper ion $Cu^{+2} \leftrightarrow Cu^{+1}$, can be inactivated by its own reaction product $H_2O_2$ accomplishing inherent peroxidatic activity (nonspecific function).There are controversial data on the occasion of ·OH radical produced[25,26,27,28]. The elevation of the ·OH radical level should proceed via Fenton-like reaction (Eq. 1).

$$Cu^{+1} + H_2O_2 \rightarrow Cu^{+2} + \cdot OH + \cdot OH^{-} \quad (1)$$

The observed increase of the ·OH radical levels in both L-41 and HLF cells at heightened total protein concentrations (2 × 0.168 mg/ml) presented in Fig. 3 can probably be explained by an above reason. It can serve as an example confirming the importance of catalase - SOD intracellular balance[17,21].

In both (L-41 and HLF) cell lines, 2 and 4 m*M* $NaN_3$ (the latter data not shown) added to the cell crude extracts (at 1 × 0.168 mg/ml and 2 × 0.168 mg/ml total protein concentrations) caused the same result. Hence, 2 m*M* $NaN_3$ is completely capable to inhibit catalase existing in the both cell crude extracts.

In the case of L-41 cells, the action of the $NaN_3$ at 2 × 0.168 mg/ml total protein concentration (that means twofold increase of catalase and SOD concentrations) caused the raising of the ·OH radical level overwhelming the model Fenton reaction level. The overwhelming of the model Fenton reaction in the case when the catalase activity is completely inhibited is an example of the Cu,Zn-SOD toxic effect.

The lack of the same effect under 3-AT used can be explained by low inhibitory capacity of 3-AT to the catalase.

As to HLF cells with lower catalase and GPx and higher Cu,Zn-SOD activities compared with L-41 cells, the $NaN_3$ action at 1 × 0.168 mg/ml and 2 × 0.168 mg/ml protein concentrations approaches ·OH radical level to the model system level (~90–93%), but does not overwhelm it.

In contrast to L-41 cells, the action of 3-AT on HLF cell crude extracts was more essential. The differed action of the both inhibitors on HLF cells can probably be connected with two reasons. Considering the ratios of $Cat_{HLF}/Cat_{L\text{-}41} = 0.38$ and $GPx_{HLF}/GPx_{L\text{-}41} = 0.65$ activities, it can be supposed that GPx contribution is higher in HLF cells. On the other hand, Cu,Zn-SOD elevated level in HLF cells can cause the ·OH radical level increase as a consequence of its peroxidatic activity. As both actions are opposite to each other, perhaps the ratio of GPx/Cu,Zn-SOD activities plays a major role in the HLF cells.

Considering the complex defensive systems of cells, it is difficult to look for a proportional dependence between enzymes activities and suppressing effects of their inhibitors, e.g., 3-AT. The generally defensive capacity of cells against $H_2O_2$ seems to depend on the ratio between catalase/GPx/SODs and $H_2O_2$, especially at high-stress situations, and the intracellular balance of these enzymes are more important than the influence of the single components.

The proposed approach enables the estimation of the cell antioxidant capacity under various stress factors without the separate measurement of the activities of the cell antioxidants. This method raises the possibility to characterize the intracellular antioxidant capacity in response to any agents and to estimate the antioxidant capacity of any new medical preparation at the cellular level.

## ACKNOWLEDGMENT

The authors would like to thank Prof. Len W. Poulter (Department of Immunology, UCL, London) for his kind gift of the HLF cell line and Dr. M. Iobadze (Institute of Medical Biotechnology, Georgian Academy of Sciences, Tbilisi) for her kind gift of the L-41 cell line used in this study. This study was supported by G-349 Grant awarded by the International Science and Technology Center (ISTC).

## BIOSKETCHES

**Tamar Kartvelishvili**, **Ph.D.,** is a Senior Researcher in the Department of Peptide Synthesis at the Institute of Molecular Biology and Biophysics (Tbilisi, Georgia). Dr. Kartvelishvili received a B.S. (in organic chemistry) in 1974 from the Tbilisi State University and a Ph.D. (in polymer chemistry) from the Institute of Organoelement Compounds (INEOS, Moscow) in 1986. Her research interests focus on the synthesis and study of new heterochain biodegradable polymers composed on naturally occurring amino acids and other nontoxic building blocks, and containing enzymatically and chemically hydrolyzable ester links; synthesis of biodegradable functional elastomers for biomedical applications; investigation of mechanisms of intracellular chromium reduction; and investigation of the cell metabolism pathways in the response of heavy metal actions. She was awarded: ISF grant (G. Soros Foundation, 1994–1995), JSPS grant (Kyoto University, 1995), research grant (Cornell University, USA), ISTC grants G-348 (2000–2002) and G-349 (2001–2003). Dr. Kartvelishvili is the author of 42 articles.

**Marina Abuladze**, **Ph.D.,** was a predoctoral fellow in the Laboratory of Cytogenecity and Cell Morphology at the Institute of Molecular Biology (Moscow). She received her doctorate in cytology from The Biological Department of Moscow State University in 1976. Currently, she is a Senior Researcher at the Andronikashvili Institute of Physics. Her work is centered on cell biology, particularly on cell proliferation and growth regulation. She was awarded ISTC grants G-348 (2000–2002) and G-349 (2001–2003). Dr. Abuladze is an author of more than 20 articles for biochemical and cell research journals.

**Nino Asatiani**, **Ph.D.,** was a predoctoral fellow in the Laboratory of Protein Research at the Institute of Molecular Biology (Moscow). She received her doctorate in biochemistry from the Institute of Plant Biochemistry (Tbilisi, Georgia) in 1985. Currently, she is a Senior Researcher at the Andronikashvili Institute of Physics. Her research efforts focus on the biochemistry of chromatin, specifically histone modification. She is awarded ISTC grants G-348 (2000–2002) and G-349 (2001–2003). Dr. Asatiani is an author of more than 20 articles for biochemical, biophysical, and molecular biology journals.

**Joseph Akhvlediani**, **Ph.D**., is a Senior Researcher in the Department of NMR at the Andronikashvili Institute of Physics (Tbilisi, Georgia). Dr. Akhvlediani received a B.S. (Physics) from the Tbilisi State University in 1961. He was predoctoral fellow in the Karpov Physical Chemistry Institute (Moscow, Russia) and received a Ph.D. from the Kazan State University (Russia) in 1969. His research interests focus on radiation physics of the solid body, superconductivity, and ESR investigation of biological systems (cells and tissues). He was awarded ISTC grants. Dr. Akhvlediani is an author of 38 articles.

**Lali Asanishvili**, received a B.S. from Tbilisi State University (Physics) in 1976. Currently, she is a Science Researcher at the Andronikashvili Institute of Physics.

**Hoi-Ying N. Holman** is a Principal Investigator at the Lawrence Berkeley National Laboratory, Berkeley, California. She received a Ph.D. in the School of Engineering, University of California at Berkeley.

**Nelly Sapojnikova**, **Ph.D.,** was a predoctoral fellow in the Laboratory of Chromatin Research at the Institute of Cytology (St. Petersburg). She received her doctorate in biophysics from the Andronikashvili Institute of Physics (Tbilisi, Georgia) in 1989. Currently, she is a Senior Researcher at the Andronikashvili Institute of Physics. Her research efforts focus on the molecular biology of chromatin, specifically the active chromatin: (1) reduced levels and altered binding of linker histone group H1; (2) acetylation of core histones; (3) the presence of HMG 1 protein. She was awarded INTAS grant (1995–1996), Wellcome Trust grant (1998–2001), and the grant from the Austrian Academy of Science (1999). Currently, she is awarded the grants from ISTC (G-348, G-349). She is an author of 23 articles for biochemical, biophysical, and molecular biology journals.
